# Through the Looking Glass: Comparing Hospitalists' and Internal Medicine Residents' Perceptions of Feedback

**DOI:** 10.7759/cureus.63459

**Published:** 2024-06-29

**Authors:** Andrew V Raikhel, Helene Starks, Gabrielle Berger, Jeffrey Redinger

**Affiliations:** 1 Department of Hospital Medicine, VA (Veteran's Affairs) Puget Sound Healthcare System, Seattle Division, Seattle, USA; 2 Department of General Internal Medicine, University of Washington, Seattle, USA; 3 Department of Bioethics and Humanities, University of Washington, Seattle, USA; 4 Department of Medicine, University of Washington School of Medicine, Seattle, USA

**Keywords:** internal medicine residency, formative feedback, feedback gap, academic hospitalist, faculty feedback

## Abstract

Introduction: Feedback is critical for resident growth and is most effective when the relationship between residents and attendings is collaborative, with shared expectations for the purpose, timing, and manner of communication for feedback. Within internal medicine, there is limited work exploring the resident and hospitalist perspectives on whether key elements are included in feedback sessions.

Methods: We surveyed internal medicine residents and supervising hospitalists at a large urban training program about their perspectives on four components of effective feedback: specificity,timeliness, respectful communication, and actionability.

Results: We received surveys from 130/184 internal medicine residents and 74/129 hospitalists (71% and 57% response rate, respectively). Residents and hospitalists differed in their perspectives about specificity and timeliness: 54% (70/129) of residents reported they did not receive specific feedback while 90% (65/72) of hospitalists reported they delivered specific feedback (p<0.01), and 33% (43/129) of residents compared with 82% (59/72) of hospitalists perceived feedback as timely (p<0.01). Internal medicine residents and hospitalists reported concordant rates of feedback sessions consisting of a two-way conversation (84%, 109/129; 89%, 64/72, respectively, p=0.82) and that communication was delivered in a respectful manner (95%, 122/129; 97%, 70/72, respectively, p=0.57).

Conclusions: We observed discordance between internal medicine residents and supervising hospitalist perspectives on the inclusion of two critical components of feedback: specificity and timing. The hospitalist cohort reported delivering more components of effective feedback than the resident cohort reported receiving. The etiology of this discordance is likely multifactorial and requires further investigation.

## Introduction

Over the past decade, graduate medical education (GME) has transitioned from a time-based format to a competency-based system for assessing readiness for independent clinical practice [1]. In this paradigm, feedback has emerged as a critical element of learner growth and development [2,3]. Recognizing the importance of feedback in professional development, the American Council of Graduate Medical Education has invested substantial time and effort to develop frameworks for delivering feedback and mandating feedback as a core component in training programs [4]. While these developments have spurred the need to critically evaluate feedback, there is a lack of consensus on how to evaluate the quality of feedback and weigh resident and attending physician perspectives [5]. Understanding differences in these perspectives is vital as evidence suggests that a collaborative relationship between resident and attending forms the foundation of effective feedback delivery [6,7]. Previous work has focused largely on only one of these perspectives from either residents or attendings [8]. Understanding both perspectives together offers an opportunity to identify areas of synergy and discord within a learning community and areas for growth in developing collaborative feedback dynamics between residents and attendings.

To gain a more nuanced understanding of the current state of feedback, we surveyed internal medicine residents (IMRs) and their hospitalist faculty supervisors at three hospitals in a large urban residency program. There is limited work in other medical specialties on how learners and teachers within a single educational environment perceive the quality and value of different feedback components [9-11]. Understanding these perspectives and how they align or differ within the context of internal medicine (IM) and hospital medicine will provide meaningful foundations for future interventions to improve the delivery and reception of feedback.

The data presented in this manuscript was presented at the Society of General Internal Medicine Northwest Chapter meeting on March 8th, 2024. 

## Materials and methods

Survey development and usability testing

We developed parallel surveys for IMRs and hospitalists based on reviews of previously published feedback surveys [12,13], feedback assessment tools, review of current literature, and discussions with local GME experts on best practices in delivering feedback. The surveys were tested for usability and clarity with representative individuals from the sample cohorts. The surveys were iteratively edited throughout this process. Both surveys included 12 questions across five components of feedback: skill/training; specificity about suggestions and behaviors [14-16]; timeliness; collaborative, considerate, and respectful communication [17]; and actionability [18-20]. These categories of questions were based on established best practices in effective feedback delivery [6,7]. These questions used a 5-point Likert scale (strongly disagree to strongly agree) to assess perceptions of effective feedback. The resident survey included an additional question about their intention to implement an action plan.

Surveys included instructions for residents to exclude training experiences in which they were supervised by non-IM faculty including emergency medicine and neurology rotations and rotations at non-academic medical centers. Hospitalists were asked to exclude experiences with medical students or visiting residents from other training programs. Both surveys also collected demographic information about gender and racial identity; residents were also asked about their year in training and hospitalists were asked about the number of years since completing training and the number of weeks per year spent with residents in their clinical work. Hospitalists were asked to report when and how they delivered their feedback. The surveys also included a reverse-scored attention check question used to exclude surveys with incorrect responses.

Participant recruitment and survey distribution

All 184 IMRs in the Seattle-based University of Washington (UW) residency program were eligible and received an email from the IMR listserv with a link to the survey. To incentivize resident survey participation, a $50 gift card was randomly awarded to four residents who completed the survey. Permission was obtained from the residency program director to survey trainees and IMR survey data was collected during May and June 2023. Eligible faculty included hospitalists and nocturnists within the UW Hospital Medicine program who supervise IMR physicians on general inpatient medicine rotations at least one week per year. The faculty survey was emailed to the 129 hospitalists who supervise IMRs on tertiary care general medicine inpatient rotations at an academic center, a county safety net hospital, and a Veterans Affairs hospital. A $100 gift card was randomly awarded to one hospitalist who completed the survey to incentivize hospitalist survey completion. Hospitalist survey data was collected during August and September 2023. Responses were confidential and de-identified after the provision of gift card incentives. The study was deemed exempt from oversight by our Institutional Review Board.

Analysis

We used descriptive statistics to summarize participant characteristics and chi-square statistics to examine differences in the proportions of residents and faculty responses after dichotomizing the Likert responses as neutral/disagree vs. agree. Analyses were performed using Microsoft Excel®.

## Results

We received surveys from 130/184 IMRs (71% response rate). One resident response was excluded based on their answer to the attention check question, thus 129 resident surveys were included in the final analysis. Among academic hospitalists, we received 74/129 surveys (57% response rate). Two hospitalists were excluded as they reported having no teaching attending weeks with residents. Demographic data for the IMR and hospitalist cohorts are reported in Table 1. 

**Table 1:** Internal medicine resident physician demographics

Gender Identity	Residents (n=129)	Attendings (n=72)
Woman	68 (55%)	41 (57%)
Nonbinary	3 (2%)	1 (1%)
Man	53 (43%)	30 (42%)
Missing/Prefer not to answer	5 (4%)	0 (0%)
Racial or ethnic identity		
Asian	40 (31)	8 (11%)
Black or African American	7 (5%)	4 (6%)
Hispanic, Latinx, or of Spanish Origin	6 (5)	1 (1%)
Native Hawaiian or Pacific Islander	1 (1%)	1 (1%)
South Asian	1 (1%)	7 (10%)
White or Caucasian	60 (47%)	47 (65%)
Other	5 (4%)	5 (7%)
Missing/Prefer not to answer	9 (7%)	8 (11%)
Resident training year		
PGY-1	44 (34%)	
PGY-2	32 (25%)	
PGY-3	39 (30%)	
PGY-4	9 (7%)	
Missing/Prefer not to answer	5 (4%)	
Attending practice site		
Hospital 1		21 (29%)
Hospital 2		29 (40%)
Hospital 3		20 (28%)
Prefer not to answer		2 (3%)
Attending years since completed medical training		
1-2		11 (15%)
3-5		16 (22%)
6-10		21 (29%)
11-20		17 (24%)
21+		3 (4%)
Prefer not to answer		4 (6%)
Years worked as teaching attending physician		
1-2		16 (22%)
3-5		15 (21%)
6-10		21 (29%)
11-20		14 (19%)
21+		2 (3%)
Prefer not to answer		4 (6%)

There was broad agreement between both IMRs (100%, 129/129) and hospitalists (99%, 71/72) that delivering effective feedback is an important skill for faculty to develop. About a third of IMRs (38%, 49/129) compared with about two-thirds of hospitalists (65%, 47/72) reported receiving training on how to effectively solicit feedback from attendings or deliver effective feedback to learners, respectively. IMRs and hospitalists also shared similar views that feedback sessions happened as two-way conversations (84%, 109/129; 89%, 64/72, respectively, p=0.82) and that communication was delivered in a respectful manner (95%, 122/129; 97%, 70/72, respectively, p=0.57). While only 56% (72/129) of IMRs reported that action plans discussed during feedback were achievable, 83% (107/129) reported an intention to act on them.

There were three areas of difference between IMRs and hospitalists (p<0.01 for all comparisons): 1) the feedback was based on directly observed, specific behaviors (71% of IMRs agreed with this statement (92/129) vs. 94% of hospitalists (68/129)); 2) the feedback itself was specific (IMRs: 54%, 70/129; hospitalists: 90%, 65/72); and 3) the feedback was shared in time to act on it (IMRs: 33%, 43/129; hospitalists: 82%, 59/72). 

Table 2 includes the complete survey results for both cohorts. Subgroup analysis of hospitalists by number of weeks per year working with residents, number of years working at a training institution, or hospital site did not reveal consistent correlations or statistically significant patterns. Hospitalists all reported using oral feedback instead of written feedback with their learners, either in a formal sit-down session at the end of working with the learner (40%), immediately after observing a task (32%), or a combination of both a formal sit-down and immediate feedback after observation (28%).

**Table 2:** Resident and hospitalist perspectives on feedback components

Feedback Components	IM Residents (Receive Feedback) N (%) Agree	Hospitalists (Give Feedback) N (%) Agree	p-Value
Feedback skills/training			
It is important for faculty to develop the skill of delivering effective feedback to learners	124 (96%)	71 (99%)	-
I have received training on how to receive/give feedback	49 (38%)	47 (65%)	-
Specific about suggestions and behaviors			
The feedback I have received/given typically includes specific suggestions on how to improve my/learner's performance	70 (54%)	65 (90%)	<0.01
The feedback I have received/given typically is based on specific behaviors that were directly observed	92 (71%)	68 (94%)	<0.01
The feedback I have received/given typically focuses on tasks or behaviors, rather than focusing on traits of personality	84 (65%)	68 (94%)	<0.01
Timely feedback			
The feedback I have received/given typically is timed as soon as possible after a task is performed	39 (30%)	59 (82%)	<0.01
The feedback I have received/given typically is given in time for me to act on it	43 (33%)	59 (82%)	<0.01
Collaborative, considerate, and respectful			
The feedback includes time to ask questions about the feedback	87 (67%)	55 (76%)	0.52
When I receive/give feedback, it is typically a two-way conversation	109 (84%)	64 (89%)	0.82
The feedback typically includes consideration of any situational challenges that were faced before giving feedback	65 (50%)	46 (64%)	0.11
The feedback I have received/given typically is delivered using respectful language	122 (95%)	70 (97%)	0.57
Actionable			
The action plan that is discussed is usually achievable	72 (56%)	47 (65%)	0.31
I typically plan to act on the action plan that was discussed during feedback sessions	107 (83%)	-	-

## Discussion

We observed considerable discordance in the perception of the timing and specificity of feedback between a broad cohort of IMRs and their supervising hospitalists. Hospitalists reported including more components of effective feedback in their delivery than the resident cohort reported receiving. This finding, consistent with previous studies in surgical programs [9,10], demonstrates considerable misalignment between the perception of feedback quality between the faculty and learner cohorts. Given the foundational role that feedback has in learner development, these findings may reflect broader discordance within the IMR and hospitalist educational alliance and may adversely impact growth opportunities for IMRs. 

Regarding the timeliness of feedback, residents largely reported that they did not receive feedback in time to act on it, while most hospitalists perceived their feedback as timely. We also observed a wide difference between the two cohorts' perceptions of whether feedback was received soon after a task was completed. Previous work has demonstrated that the ideal time for feedback delivery is context-dependent [21]; thus, learner or faculty preference, clinical context, and the nature of the task being discussed may explain differing perceptions of the optimal timing for feedback conversations among our two cohorts. Furthermore, feedback that is delivered in implicit comments, conveyed casually, or unlabeled by the hospitalist may make identification of feedback difficult [22]. Similarly, recent qualitative research demonstrates confusion surrounding the distinction between feedback and teaching among IMRs and attendings [23]. These factors illustrate that substantial barriers exist in developing a shared model for the timing of feedback. While it is unclear what underlying factors impacted the surveyed cohort, the sizable difference in perspectives indicates that further study is needed to investigate factors that impact the optimal timing of feedback.

A second major difference between the two cohorts centers on whether feedback was specific and based on directly observed behaviors. Effective feedback supports resident understanding of the difference between their recent performance and a target performance, while non-specific feedback may limit residents’ ability to navigate this gap [24]. Furthermore, feedback not based on directly observed behaviors can damage the educational alliance by suggesting improvements based on conjecture or hearsay [25]. This increases the risk that bias is introduced into feedback, amplifying existing disparities among residents with non-dominant identities [26,27]. Feedback becomes less effective and has the potential to perpetuate harm as it veers from discussing specific tasks to comments about the individual, which may reflect important aspects of identity or culture that should not be the subject of evaluation [15].

While nearly all hospitalists surveyed indicated that delivery of effective feedback is important, far fewer reported receiving training in how to deliver feedback. Additionally, IMRs reported even lower rates of training on soliciting feedback. The concordance between IMRs and hospitalists in several feedback domains, including the use of respectful language, exploration of learner perspectives, and adequate time to answer questions, would suggest that these important skills are nurtured in other aspects of faculty development, for example, in alternative trainings about cultivating a safe learning environment. Conversely, misaligned perceptions regarding timing and specificity highlight skill deficits that are more directly tied to feedback delivery. These findings likely complicate the educational alliance within a hospitalist-resident feedback dyad and suggest that targeted training on timing and specificity of feedback may help to mitigate some of the differences in residents' and hospitalists' perceptions.

The observed differences in our study may stem from differing expectations for the goals of feedback. Feedback can take many forms, including formative, summative, and corrective feedback, as well as feedback on professional behavior, among others [28,29]. These forms of feedback are each characterized by a set of best practices to support different goals. Adding further complexity is that feedback sessions may defy clear categorization as hospitalists and IMRs intuitively navigate a feedback conversation together. If expectations of the type of feedback are misaligned, for example with the IMR expecting formative feedback but receiving summative feedback, this could lead to disagreement about whether feedback was timely and based on specific, observable behaviors.

Applying our observations to the context of a feedback dyad should spur discussions early in the working relationship to develop a shared framework for the goals and context of feedback. In both formative and summative cases, aligning attending and resident perspectives on feedback requires a mutual investment of time and a shared view on how feedback should be labeled, what content areas should be included, and where and when feedback should be delivered. This mutual understanding necessitates a bidirectional educational alliance where both parties are equal partners in a feedback dialogue. Effective implementation also requires flexibility on the part of attendings to align their feedback practices with residents’ needs and the contexts of their clinical practice. On a systems level, understanding how formative and summative feedback is utilized within GME would be an important step toward aligning expectations about feedback conversations between attendings and IMRs.

Despite the observed differences, 83% of IMRs affirmed their intention to implement the feedback that they received. This finding clarifies our understanding of this cohort’s commitment to integrating feedback and is consistent with the results of prior studies of IMRs reporting a strong desire for meaningful feedback with the goal of improving proficiency. The willingness of surveyed IMRs to engage with feedback despite perceived imperfections in the content and delivery is an important finding that should inform future efforts to develop feedback training programs for faculty [30,31].

Our findings are based on faculty and learner cohorts' self-assessments of their experiences giving and receiving feedback. Self-assessment of skills is often inflated when compared with outside perspectives, consistent with our findings. Gaining an accurate understanding of the components of feedback that are delivered during feedback sessions requires methods to verify the content of the feedback exchange. This evaluation can be obtained by recording the sessions for subsequent review or through the inclusion of a third-party observer in the feedback session, both of which have received limited investigation [12,13]. These strategies present differing challenges and opportunities to investigate feedback sessions, which are typically nuanced and private, and learn more about how feedback delivered by faculty differs from the feedback perceived by the learner. 

There are several limitations to the generalizability of our findings. Social desirability bias may have affected our results by increasing rates of either IMR- or hospitalist-reported quality of feedback. Despite achieving robust response rates across three hospitals, we surveyed residents and hospitalists within a single academic system. The response rate of hospitalists also lagged that of the residents, adding some limitations to our ability to compare the two groups. Feedback is influenced by cultural elements that differ in various institutions and training centers. Data collected by researchers not directly involved in a learning community that was being studied should be able to mitigate some of the potential bias encountered. Expanding the cohort of surveyed learners and teachers to include a variety of training sites, including outpatient contexts, would increase the generalizability of the results and provide direction for future research.

## Conclusions

We observed considerable discordance between IMRs and supervising hospitalists in their perspectives regarding the timing and specificity of feedback. The surveyed cohort reported concordance in feedback consisting of a two-way conversation and using respectful language. The etiology of these findings is likely multifactorial and merits further investigation to support the development of healthy learning environments with aligned expectations regarding how feedback should be labeled, what content areas should be included, and where and when feedback should be delivered.
